# Supplementary material for: Transcriptomic Insights into Mechanisms of Early Seed Maturation in the Garden Pea (Pisum sativum L.)
Source: Cells. 2020 Mar 23;9(3):779. doi: 10.3390/cells9030779 (PMC7140803; doi:10.3390/cells9030779)
Supplement: Supplementary file 1 [file cells-09-00779-s001.zip › Supplementary materials/FileS4.zip!/FileS3/TFs - Results/Summary Sheets - Modules/Underexpression Spots/Report.pdf]

Underexpression Spots

landscape

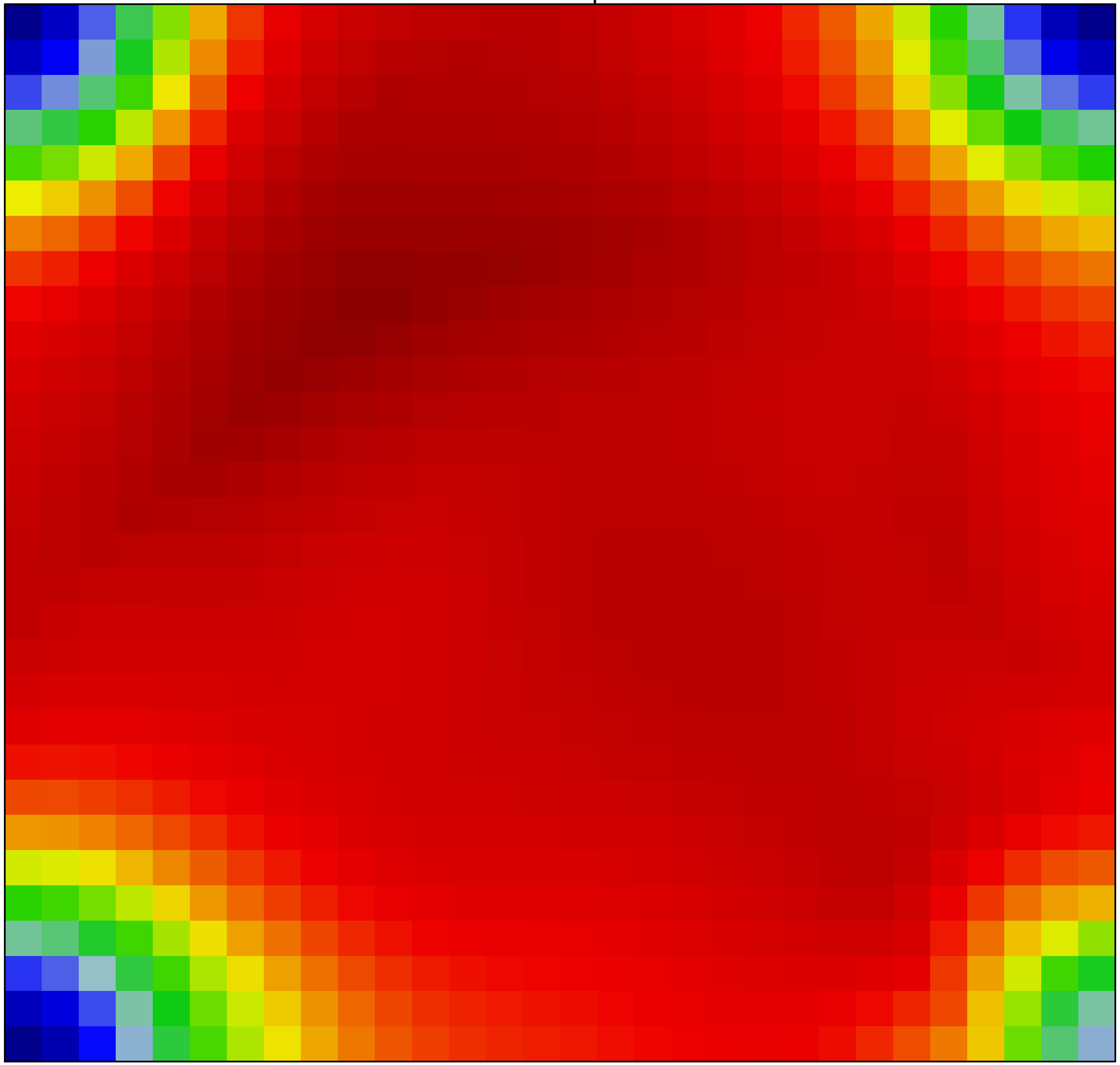

Underexpression Spots

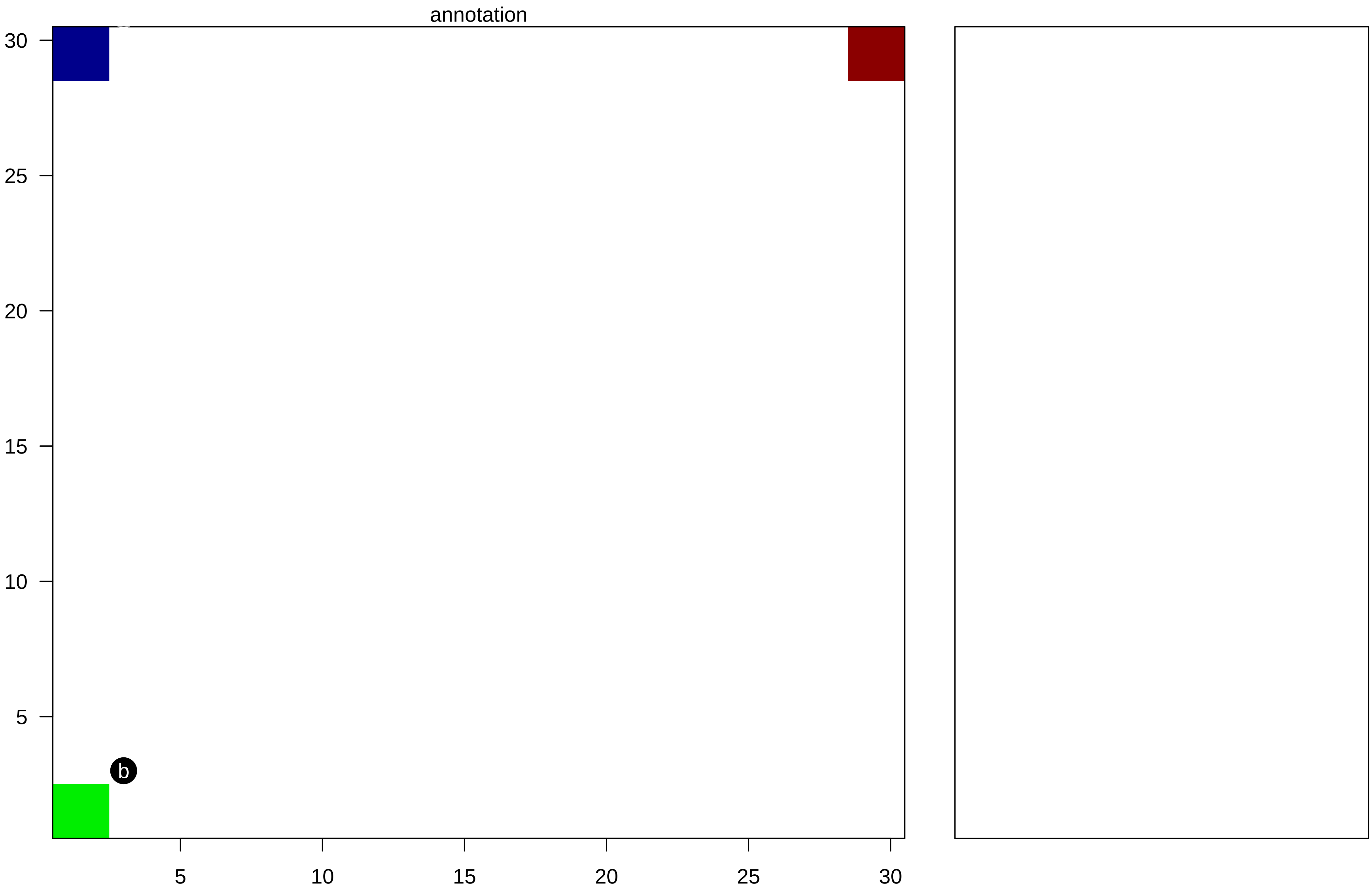

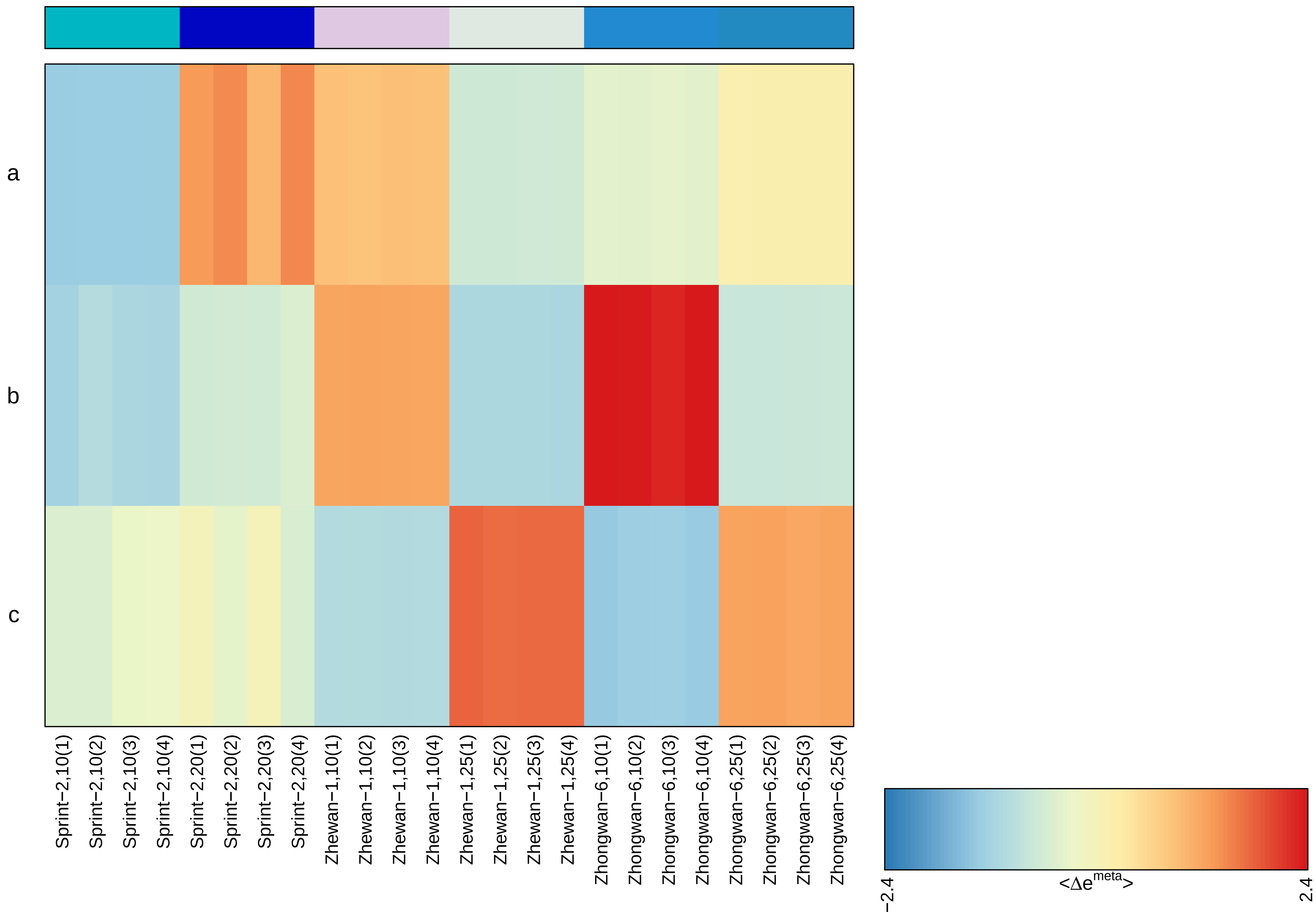

a
b
c
Sprint−2,10(1)
Sprint−2,10(2)
Sprint−2,10(3)
Sprint−2,10(4)
Sprint−2,20(1)
Sprint−2,20(2)
Sprint−2,20(3)
Sprint−2,20(4)
Zhewan−1,10(1)
Zhewan−1,10(2)
Zhewan−1,10(3)
Zhewan−1,10(4)
Zhewan−1,25(1)
Zhewan−1,25(2)
Zhewan−1,25(3)
Zhewan−1,25(4)
Zhongwan−6,10(1)
Zhongwan−6,10(2)
Zhongwan−6,10(3)
Zhongwan−6,10(4)
Zhongwan−6,25(1)
Zhongwan−6,25(2)
Zhongwan−6,25(3)
Zhongwan−6,25(4)
−2.4
<Δe^meta>
2.4

Underexpression Spots

Spot Summary: a

metagenes = 4
genes = 41

<r> metagenes = 1
<r> genes = 0.66
beta: r2= 2.67 / log p= −Inf

samples with spot = 4 (16.7 %)
Sprint−2(10) : 4 (100 %)

Overview Map

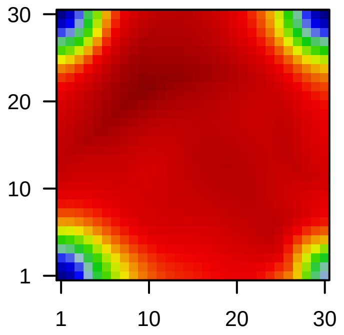

Spot

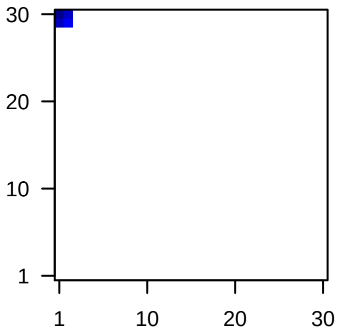

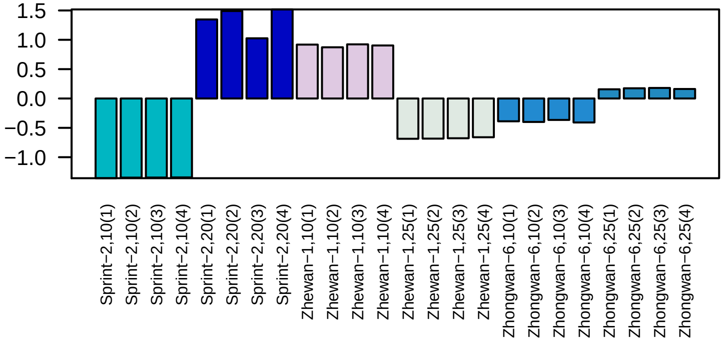

Spot Genelist

Rank	ID	max e	min e	r	Symbol	Description
1	TRINITY_DN4	8.82	−8.66	0.93		
2	TRINITY_DN4	8.39	−5.76	0.93		
3	TRINITY_DN4	5.37	−4.32	0.97		
4	TRINITY_DN6	1.32	−2.84	0.65		
5	TRINITY_DN1	3.77	−2.56	0.81		
6	TRINITY_DN5	2.36	−2.06	0.83		
7	TRINITY_DN5	3.42	−2.06	0.86		
8	TRINITY_DN4	2.18	−1.82	0.81		
9	TRINITY_DN3	3.65	−1.82	0.74		
10	TRINITY_DN5	2.24	−1.8	0.87		
11	TRINITY_DN2	2.61	−1.75	0.8		
12	TRINITY_DN1	1.71	−1.55	0.65		
13	TRINITY_DN1	1.43	−1.3	0.95		
14	TRINITY_DN5	1.1	−1.3	0.75		
15	TRINITY_DN1	1.26	−1.2	0.49		
16	TRINITY_DN5	1.6	−1.18	0.56		
17	TRINITY_DN4	1.85	−1.11	0.75		
18	TRINITY_DN4	2.8	−1.11	0.77		
19	TRINITY_DN2	2.08	−1.08	0.91		
20	TRINITY_DN1	1.73	−1.06	0.87		

Underexpression Spots

Spot Summary: b

metagenes = 4
genes = 49

<r> metagenes = 1
<r> genes = 0.75
beta: r2= 8.49 / log p= −Inf

samples with spot = 2 (8.3 %)
Sprint−2(10) : 2 (50 %)

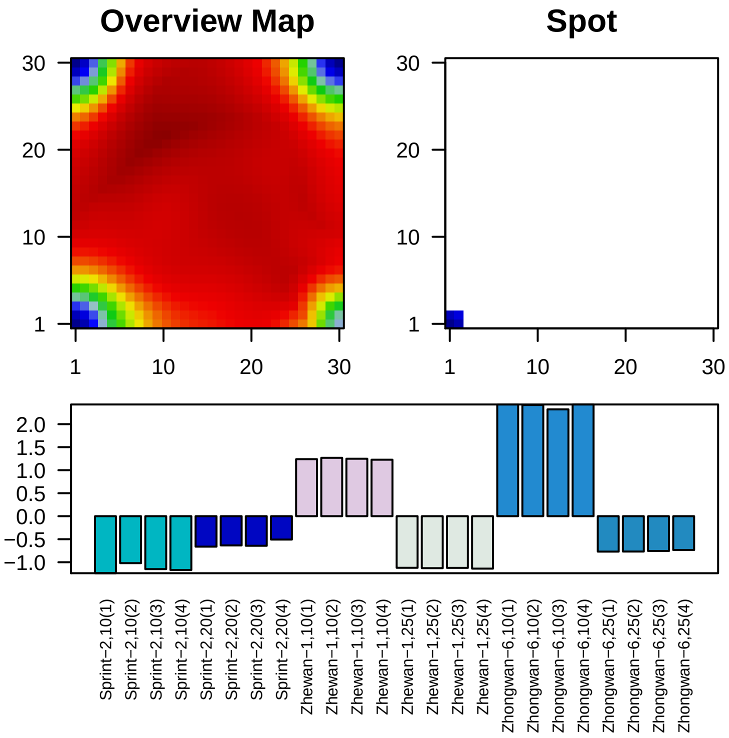

Spot Genelist

Rank	ID	max e	min e	r	Description	Symbol
1	TRINITY_DN3	3.87	−6.3	0.73		
2	TRINITY_DN1	13.12	−4.78	0.74		
3	TRINITY_DN4	5.77	−4.28	0.73		
4	TRINITY_DN1	6.17	−3.24	0.94		
5	TRINITY_DN1	3.26	−3.12	0.74		
6	TRINITY_DN1	8.39	−3.05	0.72		
7	TRINITY_DN7	11.07	−3.03	0.95		
8	TRINITY_DN4	2.64	−2.95	0.56		
9	TRINITY_DN1	5.25	−2.61	0.94		
10	TRINITY_DN3	4.41	−2.6	0.97		
11	TRINITY_DN2	2.65	−2.58	0.8		
12	TRINITY_DN3	5.76	−2.29	0.92		
13	TRINITY_DN5	2.05	−1.82	0.82		
14	TRINITY_DN1	4.57	−1.48	0.97		
15	TRINITY_DN3	2.08	−1.47	0.9		
16	TRINITY_DN1	2.87	−1.41	0.58		
17	TRINITY_DN7	3.48	−1.29	0.99		
18	TRINITY_DN3	2.03	−1.17	0.81		
19	TRINITY_DN2	4.53	−1.15	0.85		
20	TRINITY_DN1	2.25	−1.12	0.9		

Underexpression Spots

Spot Summary: c

metagenes = 4
genes = 46

<r> metagenes = 1
<r> genes = 0.68
beta: r2= 4.93 / log p= −Inf

samples with spot = 4 (16.7 %)
Zhongwan−6(10) : 4 (100 %)

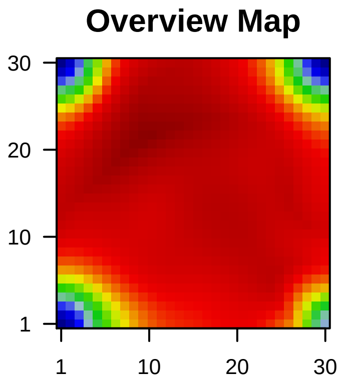

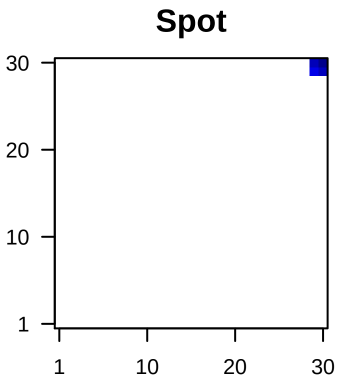

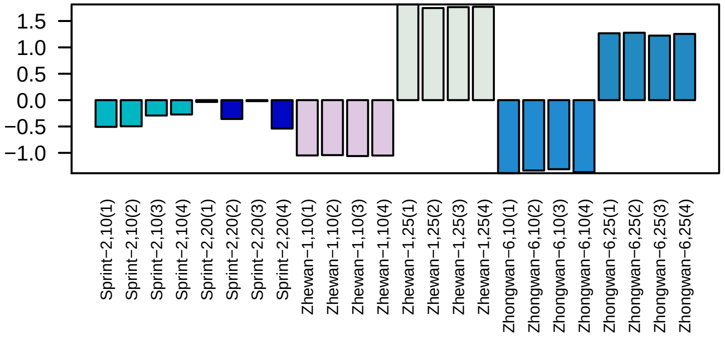

Spot Genelist

Rank	ID	max e	min e	r	Symbol	Description
1	TRINITY_DN4	7.91	−7.93	0.94		
2	TRINITY_DN4	7.74	−4.91	0.79		
3	TRINITY_DN4	1.92	−4.32	0.66		
4	TRINITY_DN2	4.81	−3.98	0.95		
5	TRINITY_DN1	7.72	−3.91	0.79		
6	TRINITY_DN7	2.62	−3.66	0.55		
7	TRINITY_DN2	4.11	−3.63	0.9		
8	TRINITY_DN3	3.31	−3.6	0.55		
9	TRINITY_DN1	10.4	−3.02	0.81		
10	TRINITY_DN5	4.22	−2.39	0.98		
11	TRINITY_DN8	2.99	−2.22	0.84		
12	TRINITY_DN2	5.9	−1.92	0.78		
13	TRINITY_DN1	2.09	−1.85	0.74		
14	TRINITY_DN2	1.66	−1.66	0.92		
15	TRINITY_DN1	1.61	−1.58	0.77		
16	TRINITY_DN1	3.23	−1.54	0.92		
17	TRINITY_DN2	2.25	−1.48	0.57		
18	TRINITY_DN4	1.01	−1.38	0.54		
19	TRINITY_DN7	1.47	−1.31	0.87		
20	TRINITY_DN2	1.22	−1.23	0.76		
